# Continuous Light Does Not Affect Atherosclerosis in APOE*3-Leiden.CETP Mice

**DOI:** 10.1177/0748730420951320

**Published:** 2020-09-11

**Authors:** Maaike Schilperoort, Rosa van den Berg, Claudia P. Coomans, Padmini P. S. J. Khedoe, Ashna Ramkisoensing, Sanne Boekestijn, Yanan Wang, Jimmy F. P. Berbée, Johanna H. Meijer, Nienke R. Biermasz, Patrick C. N. Rensen, Sander Kooijman

**Affiliations:** * Department of Medicine, Division of Endocrinology, Leiden University Medical Center, Leiden, the Netherlands; † Einthoven Laboratory for Experimental Vascular Medicine, Leiden University Medical Center, Leiden, the Netherlands; ‡ Department of Molecular Cell Biology, Division of Neurophysiology, Leiden University Medical Center, Leiden, the Netherlands; § Department of Pulmonology, Leiden University Medical Center, Leiden, the Netherlands; || Oncode Institute, Utrecht, the Netherlands; ¶ Department of Medical Oncology, Leiden University Medical Center, Leiden, the Netherlands; # Department of Endocrinology, the First Affiliated Hospital of Xi’an Jiaotong University, Xi’an, China

**Keywords:** artificial light, biological clock, cardiovascular disease, atherosclerosis

## Abstract

Artificial light exposure is associated with dyslipidemia in humans, which is a major risk factor for the development of atherosclerotic cardiovascular disease. However, it remains unclear whether artificial light at night can exacerbate atherosclerosis. In this study, we exposed female APOE*3-Leiden.CETP mice, a well-established model for human-like lipid metabolism and atherosclerosis, to either a regular light-dark cycle or to constant bright light for 14 weeks. Mice exposed to constant light demonstrated a minor reduction in food intake, without any effect on body weight, body composition, or the weight of metabolic organs. Constant light increased the plasma levels of proatherogenic non–high-density lipoprotein (HDL) cholesterol but did not increase the size or severity of atherosclerotic lesions in the aortic root. Mice exposed to constant light did show lower immune cell counts, which could explain the absence of an effect of atherosclerosis despite increased non–HDL cholesterol levels. Behavioral analysis demonstrated variability in the response of mice to the light intervention. Constant light completely blunted behavioral rhythms in some mice, while others extended their behavioral period. However, rhythm strength was not an important determinant of atherosclerosis. Altogether, these results demonstrate that constant bright light does not affect atherosclerosis in APOE*3-Leiden.CETP mice. Whether artificial light exposure contributes to cardiovascular disease risk in humans remains to be investigated.

The availability of artificial light has allowed our society to develop a 24/7 culture, thereby accelerating productivity in the workplace and providing new social opportunities. However, these advantages have come at the cost of our biological rhythm. Artificial light exposure results in misalignment between the biological clock and behavior, which causes internal desynchrony and disruption of essential physiological processes within the body (Blume et al., 2019). Indeed, artificial light at night has been associated with deleterious health effects in humans (Fonken and Nelson, 2011), such as an increased risk of developing cancer (Kloog et al., 2008; Kloog et al., 2009; Garcia-Saenz et al., 2018), psychiatric disorders (Bedrosian and Nelson, 2013), diabetes (Obayashi et al., 2014), and obesity (McFadden et al., 2014; Koo et al., 2016; Rybnikova et al., 2016; Park et al., 2019). Many of these associations have been substantiated by studies in rodents exposed to constant light, as a model of circadian disruption by artificial light at night in humans (Emmer et al., 2018).

Aside from an increased body weight, aberrant lipid profiles have been reported in humans exposed to artificial light at night (Obayashi et al., 2013). This is concerning because dyslipidemia is a major risk factor for the development of atherosclerotic cardiovascular disease, which is currently the number one cause of death globally (Benjamin et al., 2018). Dyslipidemia is most often manifested by an elevation of circulating triglycerides and proatherogenic non–high-density lipoprotein (HDL) cholesterol, which together drive the development of atherosclerotic lesions in the vessel wall (Miller, 2009). In addition to lipids and cholesterol, a proinflammatory state importantly contributes to atherosclerosis development (Hansson and Hermansson, 2011), but little is known about the effect of artificial light exposure on the immune system in humans.

Considering the above-mentioned evidence, it is tempting to assume that increased light exposure exacerbates atherosclerotic cardiovascular disease, which could have major public health consequences. This is supported by a recent epidemiological study reporting a significant association between light at night and progression of subclinical carotid atherosclerosis in an elderly population (Obayashi et al., 2019). However, whether artificial light is a causal risk factor for atherosclerosis remains to be confirmed.

In this study, we aimed to investigate whether continuous artificial light exposure aggravates atherosclerosis development in a well-established mouse model of human atherosclerosis.

## Materials and Methods

### Experimental Animals

Mice heterozygous for the APOE*3-Leiden gene (van den Maagdenberg et al., 1993) were crossbred with mice homozygously expressing human cholesteryl ester transfer protein (CETP) to yield heterozygous APOE*3-Leiden.CETP transgenic mice (Westerterp et al., 2006), a mouse model with a human-like lipoprotein metabolism. Female APOE*3-Leiden.CETP mice aged 8 to 12 weeks were group housed at 21 °C (*n* = 4-5/cage) and fed ad libitum with a Western-type diet (WTD) containing 15% (w/w) fat from cocoa butter and 1% (w/w) fat from corn oil (diet T; Hope Farms BV, Woerden, the Netherlands), enriched with 0.1% (w/w) cholesterol. We specifically used female mice for this study, as the development of hypercholesterolemia and atherosclerosis in the APOE*3-Leiden.CETP model is restricted to the female sex (Pouwer et al., 2019). During a run-in period of 3 weeks, mice were housed under standard 12 h:12 h light-dark (LD) conditions. Afterward, mice were divided into 2 groups (*n* = 18/group, each group consisting of mice from 12 different litters) that were balanced for fasting plasma total cholesterol and triglyceride levels, body weight, and age. The groups were exposed to either regular 12 h:12 h LD cycles or to 12 h:12 h light-light (LL; i.e., constant bright-light exposure) for the total duration of 14 weeks. During the last 10 days of the light intervention, behavioral activity patterns were assessed by housing mice individually in cages fitted with passive infrared detectors. At the end of the study, mice were killed by cervical dislocation around 1000 h, corresponding to zeitgeber time (ZT) 2 (i.e., 2 h after onset of the light phase) of the control group, and tissues (i.e., heart, aorta, liver, spleen, bone marrow, interscapular brown adipose tissue [iBAT], gonadal white adipose tissue [gWAT], and subcutaneous white adipose tissue [sWAT]) were collected for further analyses.

A second cohort of WTD-fed female APOE*3-Leiden.CETP mice aged 8 to 12 weeks was group housed (*n* = 3-5/cage) and, after a 3-week run-in period, subjected to either LD or weekly alternating light dark cycles (12-h shifts; LD-DL) for the total duration of 15 weeks, as described previously (Schilperoort et al., 2020). After 10 weeks, a subset of mice was killed by CO_2_ inhalation at ZT0 and ZT12 (*n* = 7-8 per timepoint/group) on the third day, after a switch in light regime to collect aortas for gene expression analysis. During week 14 and 15 of the light intervention, behavioral activity patterns were assessed by housing mice (*n* = 15/group) individually in cages fitted with passive infrared detectors.

Mice were housed in clear plastic cages with minimal and semitranslucent bedding, placed in light-tight cabinets fitted with diffuse white fluorescent light (~100 lux). The light intensity and spectral power distribution of the light source were evaluated using an AvaSpec 2048-SPU light meter (Avantes BV, Apeldoorn, the Netherlands; Suppl. Fig. S1). All mouse experiments were approved by the institutional ethics committee on animal care and experimentation at Leiden University Medical Center, Leiden, the Netherlands.

### Food Intake, Body Weight, and Body Composition Measurements

Food intake and body weight were measured throughout the study at 1200 h, corresponding to ZT4 of the control group. Food intake was monitored at weekly intervals by weighing food on the lid of the cages. During the first 12 weeks of the study, food intake was determined per cage in group-housed animals (*n* = 4-5/cage). During the last 2 weeks of the study period, food intake was determined in individually housed animals. Every 2 to 4 weeks, body weight was measured with a scale. At the end of the study, after 14 weeks, body composition was measured with an EchoMRI-100-analyzer (EchoMRI, Houston, TX).

### Plasma Lipid Measurements

Every 2 to 4 weeks, unfasted blood samples were obtained at 1200 h, corresponding to ZT4 of the control group, for plasma lipid measurements. Plasma was isolated by centrifugation, and plasma triglyceride and total cholesterol levels were measured by using enzymatic kits (Roche Diagnostics, Indianapolis, IN). HDL was isolated by precipitation of ApoB-containing lipoproteins, wherein 20% polyethylene glycol 6000 (Sigma-Aldrich, St. Louis, MO) in 200 mM glycine-buffered saline (pH 10) was added to plasma (1:2, v/v), mixed, and centrifuged for 30 min at 6000 rpm. HDL cholesterol was determined by measuring the total cholesterol in the supernatant as described above. Non–HDL cholesterol was calculated by subtracting the HDL cholesterol from the total cholesterol in plasma.

### Immune Cell Composition

At the end of the study, after 14 weeks of LD or LL intervention, mice were anesthetized by intraperitoneal injection of acepromazine (6.25 mg/kg; Sanofi Santé Nutrition Animale, Libourne, France), midazolam (6.25 mg/kg; Roche, Basel, Switzerland), and fentanyl (0.31 mg/kg; Janssen-Cilag BV, Breda, the Netherlands), and blood was drawn retro-orbitally into EDTA-coated cups for immune cell analysis. After the mice were killed, long bones were removed and bone marrow was flushed into EDTA-coated cups. The immune cell composition of freshly isolated whole blood and bone marrow was determined using a Sysmex XT-2000i hematology analyzer (Sysmex Corporation, Kobe, Japan).

### Histological Analysis of the Heart

Hearts were fixated in phosphate-buffered 4% paraformaldehyde, embedded in paraffin, and cross-sectioned (5 µm) throughout the aortic valve region. Sections were stained with hematoxylin-phloxine-saffron for histologic analysis. The area of the atherosclerosis lesion was analyzed in the aortic root, starting from the appearance of the open aortic valve leaflets in 4 subsequent sections with 50-µm intervals using ImageJ software (version 1.50). Lesion severity (mild: types I-III and severe: types IV-V) was scored according to the guidelines of the American Heart Association adapted for mice, as described previously (Zadelaar et al., 2006).

### Gene Expression Analysis

RNA was extracted from iBAT, gWAT, liver, and aorta using TRIzol RNA isolation reagent (Thermo Fisher, Waltham, MA) following the manufacturer’s protocol. RNA concentration was determined with a NanoDrop spectrophotometer (Thermo Fisher), and 100 ng RNA was transcribed with M-MLV reverse transcriptase (Promega, Madison, WI). Quantitative reverse-transcriptase polymerase chain reaction was performed using a SYBR Green kit (Promega) on a 7500 Fast RT-PCR System (Applied Biosystems, Foster City, CA). Primer sequences are listed in Supplementary Table S1. The mRNA expression of genes of interest was normalized to the mRNA expression of the housekeeping gene *36b4*.

### Statistical Analysis

All data are expressed as means ± SEM, and statistical analysis was performed using GraphPad Prism (version 8.1.1). Means were compared using a 2-tailed unpaired Student *t* test, for which the test statistics are included in Supplementary Table S2. When measurements were taken over time, comparisons were made using repeated-measures analysis of variance (ANOVA) or mixed models in case of missing values (test statistics are included in Suppl. Table S3). Pearson correlation analysis was performed to examine potential linear relationships between variables. Behavioral patterns were analyzed using ClockLab data analysis software (Actimetrics, Wilmette, IL). F-periodograms were plotted using activity data binned into 10-min intervals to evaluate rhythm strength, as defined by the amplitude in the periodogram (q) and rhythm period. Actograms were plotted for visualization of behavioral patterns. Differences between groups were considered statistically significant if *p* < 0.05.

## Results

### Constant Light Mildly Reduces Food Intake without Altering Body Weight

**Figure 1.**
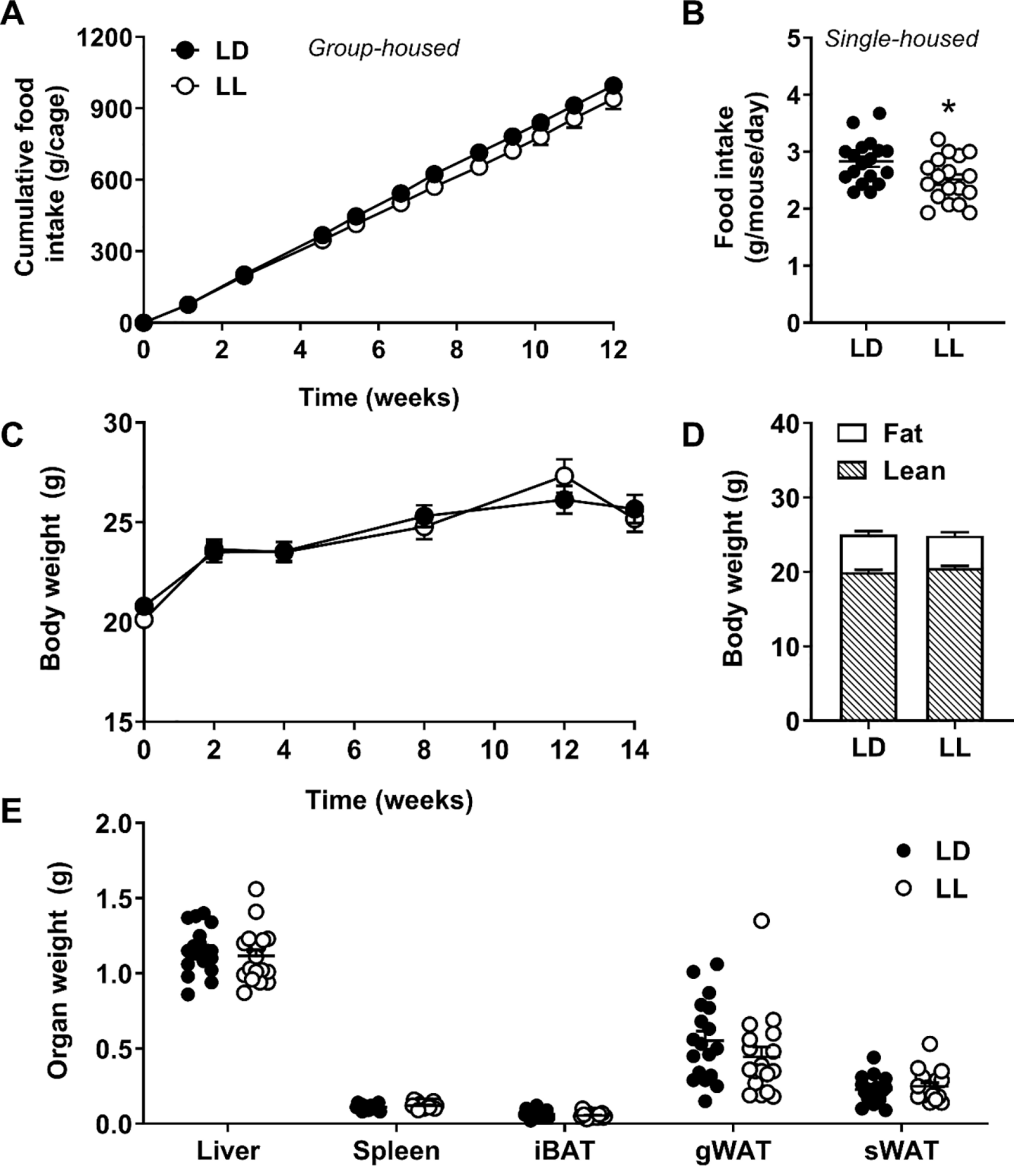
Constant light decreases food intake without affecting body weight or adiposity. APOE*3-Leiden.CETP mice fed a Western-type diet were exposed to LD or LL (*n* = 18/group). Mice were group housed up to and including week 12, and the average cumulative food intake per cage (*n* = 4) was calculated for this period (A). In weeks 13 and 14, mice were single housed, and the average food intake per mouse per day was determined (B). Body weight was measured at regular intervals (C), and body composition was determined at endpoint (D). Weights of the liver, spleen, interscapular brown adipose tissue, gonadal white adipose tissue, and subcutaneous white adipose tissue were measured at endpoint (E). Data are expressed as individual values or as means ± SEM. Significance was tested by 2-way repeated-measures analysis of variance (A, C) or 2-tailed unpaired Student *t* test (B, D, E), of which the test statistics are included in Supplementary Tables S2 and S3. *p < 0.05 compared with the LD control group. Color version of the figure is available online.

Dyslipidemic female APOE*3-Leiden.CETP mice were subjected to 14 weeks of either normal LD cycles or kept under constant LL conditions. During the first 12 weeks of the study, when mice were group housed, we observed a marginally but consistently lower food intake in mice subjected to LL (Fig. 1A). Analysis of food intake over time by 2-way ANOVA revealed a significant interaction between time and group (*F*_10,60_ = 2.253, *p* = 0.026). Although the effect of group alone was not statistically significant (*F*_1,6_ = 1.563, *p* = 0.258), possibly related to the insufficient power due to the group housing resulting in an *n* = 4 per experimental group, we observed a significantly lower food intake in the last 2 weeks of the study when mice were single housed (−11.2%; *p* = 0.020; Fig. 1B; *n* = 18 per experimental group). This difference in food intake did not result in significant differences in the body weight of mice over time (Fig. 1C) nor in fat mass or lean mass as determined by EchoMRI (hereto magnetic resonance imaging at the endpoint of the study; Fig. 1D). In addition, after 14 weeks of light intervention, weights of various metabolic organs were similar between LD and LL mice (Fig. 1E). Of note, the fact that we did not observe differences in body weight and adiposity—while this was previously demonstrated in male mice (Fonken et al., 2010; Coomans et al., 2013; Fonken et al., 2013; Kooijman et al., 2015; Lucassen et al., 2016)—may be explained by the use of female instead of male APOE*3-Leiden.CETP mice, as we found that females are less susceptible to high-fat diet-induced obesity (unpublished observation).

### Constant Light Increases Plasma Non–HDL cholesterol and Decreases White Blood Cell Counts

**Figure 2.**
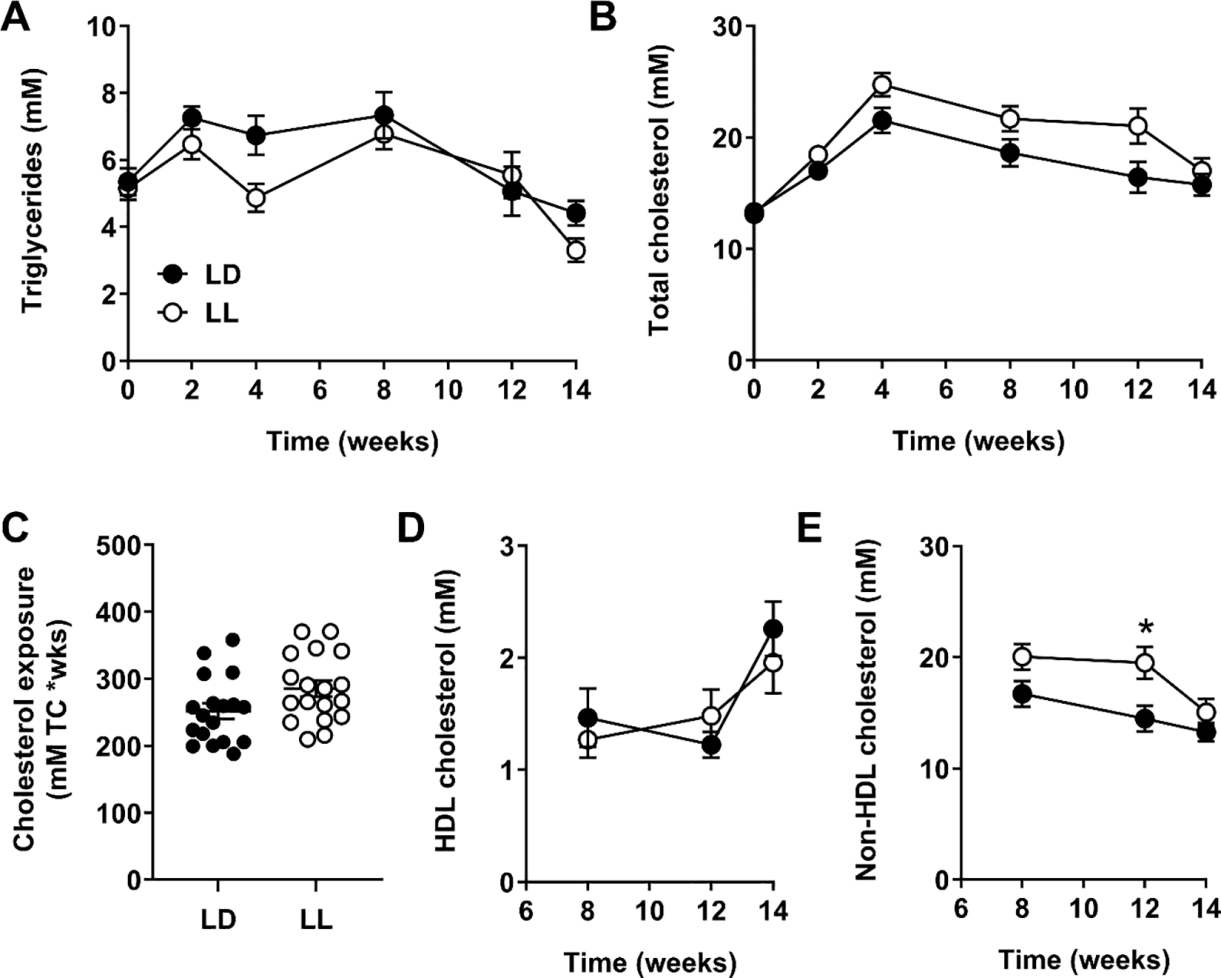
Constant light increases plasma non–high-density lipoprotein (HDL) cholesterol. APOE*3-Leiden.CETP mice fed a Western-type diet were exposed to LD or LL (*n* = 18/group), and plasma triglycerides (A) and total cholesterol (B) was measured every 2 to 4 weeks. Total cholesterol exposure throughout the study was calculated from the total cholesterol measurements (C). At weeks 8, 12, and 14, plasma HDL cholesterol (D) and non–HDL cholesterol (E) were measured. Data are expressed as individual values or as means ± SEM. Significance was tested by mixed models (A, B, D, E) or 2-tailed unpaired Student *t* test (C), of which the test statistics are included in Supplementary Tables S2 and S3. *p < 0.05 compared with the LD control group.

As circulating lipids and cholesterol are a key determinants of atherosclerosis, we monitored plasma triglycerides and total cholesterol throughout the study. While plasma triglyceride levels were not significantly different between LD and LL mice (Fig. 2A; no significant group effect nor time-by-group interaction effect by mixed models), plasma total cholesterol levels were higher over time in LL mice as compared with LD mice, as demonstrated by a significant interaction between time and group (*F*_5,158_) = 2.801, *p* = 0.019; Fig. 2B). Accordingly, cholesterol exposure, as determined by calculating the area under the curve of all individual plasma cholesterol measurements, was nonsignificantly higher in LL mice (+13%; *p* = 0.052; Fig. 2C). HDL cholesterol was identical between the groups (Fig. 2D), but non–HDL cholesterol was increased over the course of the experiment (*F*_1,33_ = 5.771, *p* = 0.022; Fig. 2E). This difference in non–HDL cholesterol was most prominent after 12 weeks of light intervention (+35%; *p* = 0.038).

**Figure 3.**
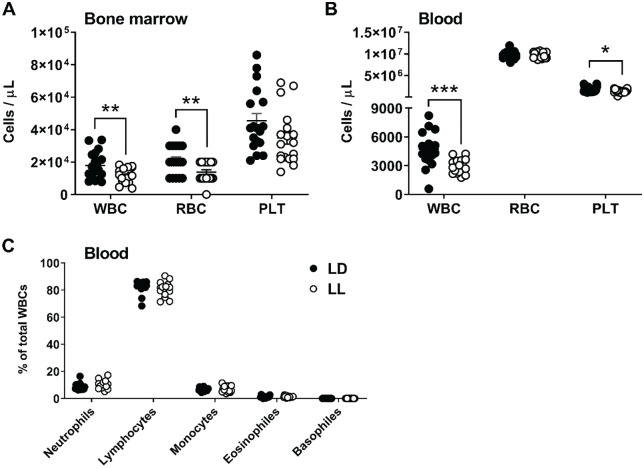
Constant light reduces the number of white blood cells in bone marrow and blood. APOE*3-Leiden.CETP mice fed a Western-type diet were exposed to LD or LL (*n* = 18/group) for 14 weeks, after which Sysmex analysis was used to evaluate the concentration of white blood cells (WBCs), red blood cells (RBCs), and platelets (PLTs) in bone marrow (A) and blood (B). In addition, different types of WBCs were quantified as a percentage of total WBCs in the blood (C). Data are expressed as individual values, including the means ± SEM. Significance was tested by the 2-tailed unpaired Student *t* test, of which the test statistics are included in Supplementary Table S2. *p < 0.05, **p < 0.01, ***p < 0.001 compared with the LD control group.

Next, we aimed to evaluate the inflammatory state as another important contributor of atherosclerosis. We used Sysmex analysis to examine the (immune) cell composition of bone marrow and blood. The bone marrow of LL mice demonstrated a lower concentration of white blood cells (−36%; *p* = 0.009) and red blood cells (−34%; *p* = 0.008; Fig. 3A). We also observed a markedly lower concentration of white blood cells in the circulation (−39%; *p* = 0.0003), along with a lower concentration of platelets (−26%; *p* = 0.013; Fig. 3B). We did not find significant differences in immune cell composition between LD and LL mice (Fig. 3C), suggesting that the lower number of white blood cells is the result of a lowering of all white blood cell types.

### Constant Light Does Not Affect Atherosclerosis

**Figure 4.**
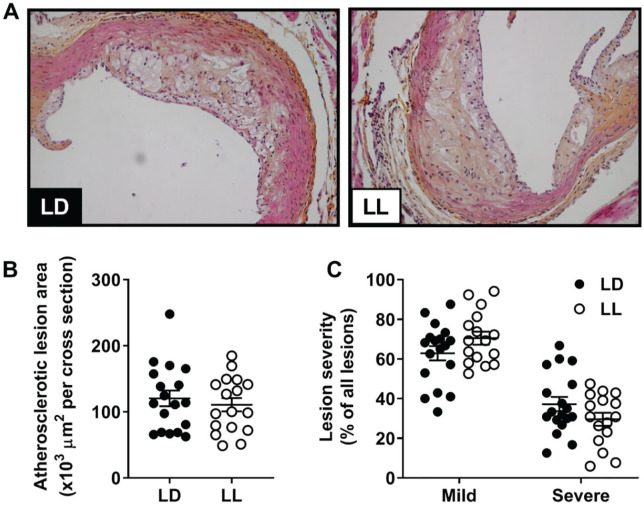
Constant light does not affect the size or severity of atherosclerotic lesions. APOE*3-Leiden.CETP mice fed a Western-type diet were exposed to LD or LL (*n* = 18/group) for 14 weeks, after which hearts were isolated and the valve area of the aortic root was stained with hematoxylin-phloxine-saffron. (A) Mean atherosclerotic lesion area was determined from 4 cross sections of the aortic root (B), and lesion severity (mild, type I-III vs severe, and type IV-V) was scored (C). Data are expressed as individual values, including the means ± SEM. Significance was tested by 2-tailed unpaired Student *t* test, of which the test statistics are included in Supplementary Table S2.

To determine whether the observed changes in cholesterol and/or immune cells affected atherosclerosis, we evaluated atherosclerotic lesions in the aortic root (Fig. 4A). Both LD and LL groups showed a comparable lesion area (Fig. 4B). In addition, lesion severity was similar between both groups, with an approximately equal number of mild (type I-III) and severe (type IV-V) lesions (Fig. 4C).

### Rhythm Strength Is Differentially Affected by Constant Light but Does Not Explain Variation in Atherosclerosis

**Figure 5.**
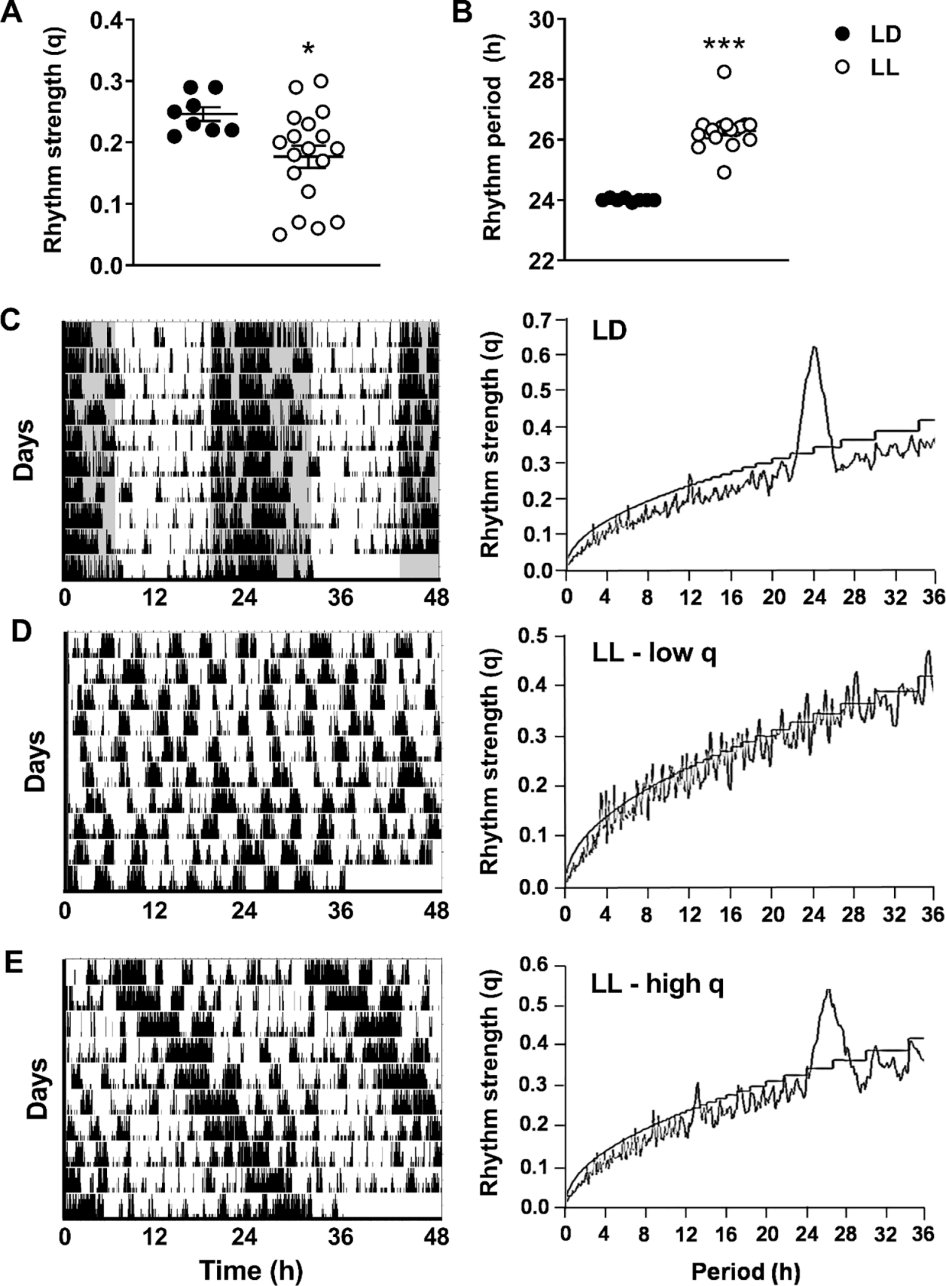
Rhythm strength is variably lower in mice exposed to continuous light. APOE*3-Leiden.CETP mice fed a Western-type diet were exposed to LD or LL (*n* = 18/group) for 14 weeks. During the last 10 study days, *n* = 8 LD mice and *n* = 18 LL mice were housed individually in cages fitted with passive infrared detectors to assess behavioral activity patterns. F-periodogram analysis was performed to calculate the rhythm strength (A) and rhythm period (B) of behavioral activity. Representative double-plotted actograms (left) and F-periodograms (right) of LD mice (C), LL mice with a low rhythm strength (low q) (D), and LL mice with a high rhythm strength (high q) (E) are shown. Data are expressed as individual values, including the means ± SEM. Significance was tested by 2-tailed unpaired Student *t* test, of which the test statistics are included in Supplementary Table S2. *p < 0.05, ***p < 0.001 compared with the LD control group.

To confirm that the light intervention disrupted circadian rhythm, we determined rhythm strength and period by evaluating patterns of behavioral activity. As expected, rhythm strength was significantly lower in LL versus LD mice (−28%; *p* = 0.022; Fig. 5A), while rhythm period was more than 2 h longer (*p* < 0.0001; Fig. 5B). When examining actograms of LD and LL mice, we observed that in contrast to LD mice, all of which showed a comparable actogram profile (Fig. 5C), actograms of LL mice demonstrated quite some variation. Some mice exposed to LL demonstrated a completely random pattern of behavioral activity as reflected by a low rhythm strength (Fig. 5D), whereas others still demonstrated a somewhat organized behavioral activity pattern with a relatively high rhythm strength and a rhythm period longer than 24 h (Fig. 5E).

**Figure 6.**
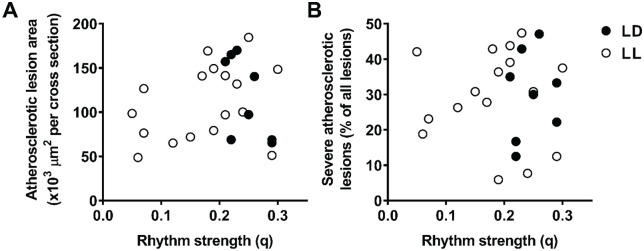
Rhythm strength does not correlate with atherosclerotic lesion area or severity. We correlated the rhythm strength of APOE*3-Leiden.CETP mice exposed to LD (*n* = 8) or LL (*n* = 18) to the atherosclerotic lesion area (A) and the relative amount of severe atherosclerotic lesions (B). Pearson correlation analysis was performed in the LD and LL groups together and separately, and the corresponding *R*^2^ and *p* values are reported in Supplementary Table S4.

Given that rhythm strength was highly variable in LL mice, we wondered whether variation in rhythm strength could explain (some of) the observed variation in atherosclerosis. However, we did not find negative correlations between rhythm strength and the atherosclerotic lesion area (Fig. 6A; Suppl. Table S4) nor between rhythm strength and the relative number of severe atherosclerotic lesions (Fig. 6B; Suppl. Table S4), indicating that rhythm strength was not a determinant of atherosclerosis in this study.

Because we have previously observed an effect of circadian disruption through repeated shifts in light-dark cycle (LD-DL) on atherosclerosis (Schilperoort et al., 2020), we evaluated whether that intervention affects rhythm strength differently from LL. Rhythm strength calculated per week was lower in LD-DL mice as compared with LD mice (−34%; *p* = 0.0009; Suppl. Fig. S2A), an effect that seemed to be more consistent between mice as compared with the LL intervention. The calculation of rhythm strength per day revealed a significant time-by-group interaction effect (*F*_9,220_ = 17.33, *p* < 0.0001), as determined by mixed models. Rhythm strength in LD-DL mice was strongly reduced, particularly 1 to 2 days after a shift in LD cycle (−55%, *p* = 0.0002 and −49%, *p* = 0.0022, respectively; Fig. S2B), which can also be appreciated from representative actograms (Suppl. Fig. S2C). Rhythm strength (per week) in LD-DL mice did not correlate to the atherosclerotic lesion area (Suppl. Fig. S2D) but negatively correlated to the atherosclerotic lesion severity (*R*^2^ = 0.368; *p* = 0.0279; Suppl. Fig. S2E).

### Constant light affects clock gene expression in peripheral tissues

**Figure 7.**
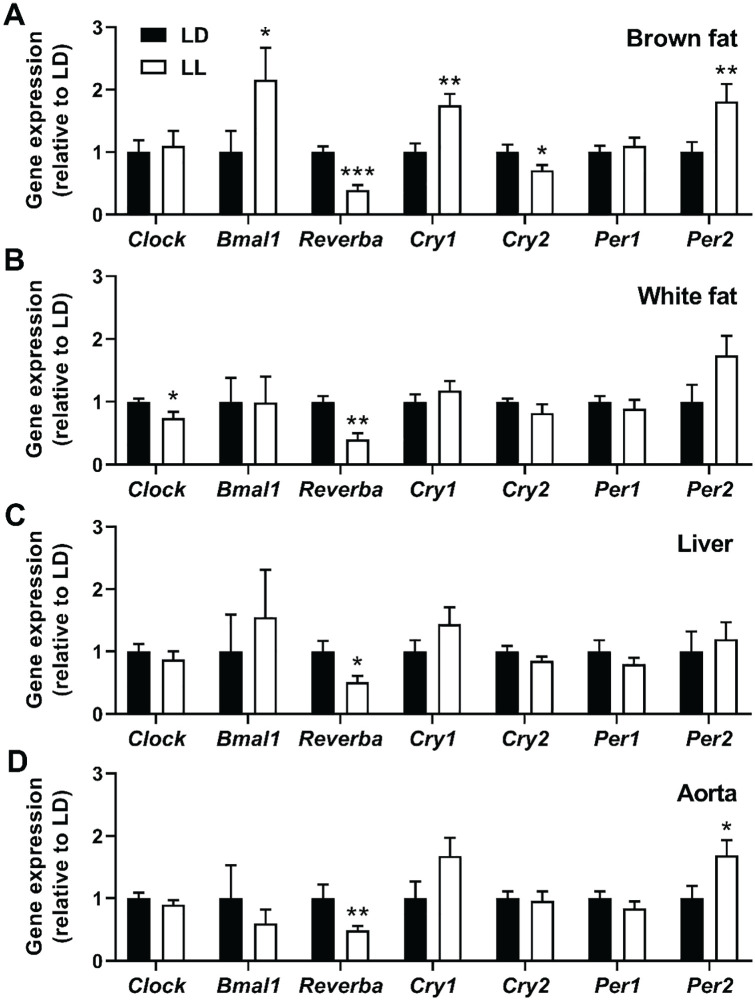
Expression of clock genes is disrupted in mice exposed to continuous light. APOE*3-Leiden.CETP mice fed a Western-type diet were exposed to LD or LL (*n* = 18/group) for 14 weeks, after which the interscapular brown adipose tissue (A), gonadal white adipose tissue (B), liver (C), and aorta (D) were isolated (*n* = 9/group) at ZT2 for analysis of clock gene expression. Data are expressed as means ± SEM. Significance was tested by 2-tailed unpaired Student *t* test, of which the test statistics are included in Supplementary Table S2. *p < 0.05, **p < 0.01, ***p < 0.001 as compared with the LD control group.

We next aimed to confirm circadian disruption in LL mice by measuring the expression of various clock genes within peripheral tissues. We could detect alterations in clock gene expression in brown fat (Fig. 7A), white fat (Fig. 7B), and liver (Fig. 7C) of LL mice as compared with LD mice, of which brown fat was most strongly affected by the light intervention (5 of the 7 measured clock genes were significantly up- or downregulated). Clock gene expression was also measured in the aortic vessel wall, which is a common site of atherosclerosis. The expression of *Reverba* and *Per2* in the aorta was significantly affected by the LL intervention (−51%, *p* = 0.006 and +69%, *p* = 0.032; Fig. 7D) but not to the same extent as observed in mice exposed to LD-DL (Suppl. Fig. S3), at least at ZT0-2. Also, although gene expression markers of inflammation, oxidative stress, and leukocyte recruitment were previously found to be upregulated in the aorta of LD-DL mice at ZT0 (Schilperoort et al., 2020), the aortas of mice exposed to LL did not show any changes in the markers *Tnfa*, *Sod1*, *Icam1*, and *Vcam1* (Suppl. Fig. S4).

## Discussion

In this study, we investigated whether continuous artificial light exposure aggravates atherosclerosis by exposing APOE*3-Leiden.CETP mice to either normal LD cycles or LL conditions. Our main findings demonstrate that constant light mildly modulates plasma cholesterol and circulating immune cells, without affecting atherosclerotic lesion size or severity.

Previously, we and others demonstrated that prolonged light exposure promotes body weight gain and the accumulation of fat mass in male wild-type mice (Fonken et al., 2010; Coomans et al., 2013; Fonken et al., 2013; Kooijman et al., 2015; Lucassen et al., 2016). In the current study, we did not find any effect of constant light on body weight or composition in female APOE*3-Leiden.CETP mice. This seeming discrepancy could potentially be explained by the use of APOE*3-Leiden.CETP instead of wild-type mice and/or by the use of female instead of male mice. It is well known that male mice are more susceptible to diet-induced obesity than female mice (Hong et al., 2009), and this also holds true for APOE*3-Leiden.CETP mice (unpublished observations). In addition, it has recently been shown that exposure to light at night increases body weight in young male but not female mice (Cisse et al., 2017), suggesting that male mice are also more susceptible to the negative metabolic effects of artificial light. Artificial light at night has been associated with obesity in cohorts of women (McFadden et al., 2014; Park et al., 2019) and cohorts of men and women combined (Reid et al., 2014; Koo et al., 2016; Rybnikova et al., 2016), but sex differences in effect size have not yet been investigated in humans. As both obesity and circadian rhythm demonstrate sex-specific characteristics (Lovejoy and Sainsbury, 2009; Santhi et al., 2016), sex differences in light-driven obesity in humans would be an interesting subject for future research.

Mice exposed to constant light showed no changes in HDL cholesterol but a mild increase in non–HDL cholesterol. We have previously shown that plasma cholesterol does not demonstrate a robust diurnal rhythm (Schilperoort et al., 2020), suggesting that the observed differences in non–HDL cholesterol are not the result of a shift in acrophase. Although non–HDL cholesterol importantly contributes to atherosclerosis development, we did not find an increase in the size or severity of atherosclerotic lesions following constant light exposure. Constant light did reduce the number of circulating leukocytes, which confirms previous findings in rodents (Bedrosian et al., 2011; Lucassen et al., 2016). The circadian clock is a potent regulator of the immune system and dictates diurnal rhythms in circulating leukocytes (Labrecque and Cermakian, 2015; Orozco-Solis and Aguilar-Arnal, 2020). In our study, samples for leukocyte measurements were obtained at a time point corresponding to ZT4 of the control group, which is around the time of the physiological circadian peak in circulating leukocytes (Scheiermann et al., 2012; Druzd et al., 2017). A lower circulating leukocyte number in mice exposed to constant light could therefore be the result of a blunted rhythm in circulating white blood cells, rather than an overall reduction in the production of white blood cells through hematopoiesis. Nevertheless, we also found a lower number of leukocytes in bone marrow of mice exposed to constant light. As bone marrow does not display a diurnal rhythm in total white blood cell counts (Stenzinger et al., 2019), these data suggest that hematopoiesis and thus overall circulating white blood cells could indeed have been reduced. Considering the essential role that immune cells play in the pathophysiology of atherosclerosis (Libby, 2002), such a reduction in leukocytes may have counteracted the proatherogenic effects of increased cholesterol levels in our study.

We have previously shown that the response of mice to a constant light intervention is variable. In some mice, constant light exposure results in severe loss of rhythmicity, whereas other mice are relatively resistant to this intervention and remain fairly rhythmic with a lengthened free-running period (Lucassen et al., 2016). These observations are confirmed in the current study and made us wonder whether a potential effect of constant light on atherosclerosis could be concealed by mice that remain rhythmic upon constant light exposure. However, this did not appear to be the case, as rhythm strength did not correlate to atherosclerosis. As of yet, it is unclear why such a variability exists in the response of mice to a constant light intervention. Nevertheless, variation has been reported in the extent of desynchronization of clock gene expression within the suprachiasmatic nucleus (SCN; Ohta et al., 2005) as well as in the robustness of rhythms in electrical activity of the SCN (Lucassen et al., 2016). In our study, variability in the rhythm of mice exposed to constant light may have been augmented by group housing. For the purpose of this study, group housing was preferred over single housing, as the latter could aggravate atherosclerosis on its own by inducing stress and hyperphagia (Schipper et al., 2018). A disadvantage of group housing is that it enables mice to huddle together, thereby providing some shelter to the light intervention. However, we previously observed negative health effects of continuous light in group-housed animals (Lucassen et al., 2016), indicating that group housing per se does not prevent the deleterious effects of increased light exposure.

Very recently, another study has been published in which effects of constant light exposure on atherosclerosis were investigated. Here, atherosclerosis-prone *Apoe*^-/-^ mice were subjected to constant light for 12 weeks, which exacerbated atherosclerosis in male but not female mice (Chalfant et al., 2020). These findings corroborate the results from our study, in which we did not find effects of constant light on atherosclerosis in a female mouse model. Similar to the potential sex differences in light-driven obesity as discussed above, these findings strongly suggest that male mice are also more susceptible to light-induced atherosclerosis progression. However, this may not be the case for other methods of circadian disruption. We have recently performed a study in which we subjected female APOE*3-Leiden.CETP mice to weekly shifts in LD cycle to disrupt circadian rhythm in a way that occurs in human shift workers. Like exposure to light at night, shift work has been associated with various metabolic disorders including cardiovascular disease (Brown et al., 2009; Vyas et al., 2012; Reutrakul and Knutson, 2015; Vetter et al., 2016). It is unclear whether these negative health effects of shift work are mainly due to aberrant light exposure or whether other factors (e.g., diminished sleep, alterations in physical activity, alterations in food intake) play an important role. In our model, we found that repeated shifts in LD cycle aggravates atherosclerosis by promoting oxidative stress and inflammation in the vessel wall, without affecting circulating levels of cholesterol or immune cells (Schilperoort et al., 2020). These results are clearly different from those of the current study, indicating differential effects of continuous versus mistimed light exposure on cardiometabolic outcome. Our data demonstrate a more profound effect of shifts in LD cycle on the circadian timing system of female mice, which could explain why that intervention, rather than constant light, aggravates atherosclerosis in female APOE*3-Leiden.CETP mice.

Not only does artificial light exposure disrupt intrinsic circadian rhythm but it also has a negative impact on sleep quality and quantity in humans, which could further exacerbate cardiovascular health (Cappuccio et al., 2011; Cho et al., 2016; Cho et al., 2018). This is supported by a recent study showing that chronic sleep fragmentation aggravates atherosclerosis in *Apoe*^-/-^ mice (McAlpine et al., 2019). Mechanistically, they found that mice subjected to sleep fragmentation produce less hypocretin, a neuropeptide that controls hematopoiesis, resulting in increased levels of neutrophils and monocytes that contribute to atherosclerosis development. This is likely the result of reduced sleep quality rather than quantity, as mice subjected to sleep fragmentation generally maintain their total sleep duration (Ramesh et al., 2009; Ramesh et al., 2012). In our study, we used a constant bright-light intervention that has been shown to disrupt circadian sleep-wake rhythms in rats (Stephenson et al., 2012). However, nocturnal rodents exposed to prolonged light also extend their sleep duration (Borbély and Neuhaus, 1978; Stephenson et al., 2012), which may compensate for a potentially reduced sleep quality. Thus, overall sleep in mice exposed to constant light may not have been impaired, which could explain why hematopoiesis and atherosclerosis were not affected in the same way as observed with sleep fragmentation.

To conclude, we demonstrate that constant light increases plasma cholesterol while decreasing circulating leukocytes in female APOE*3-Leiden.CETP mice, resulting in no net effect on atherosclerosis. In contrast, constant light has been shown to aggravate atherosclerosis in male mice, and other interventions that disturb circadian rhythm in mice, such as shifting LD cycles or chronic sleep disruption, have shown effects on atherosclerosis in both sexes via distinctive mechanisms. Thus, selection of an appropriate experimental model seems paramount when investigating the effect of circadian disruption on cardiovascular health. By using our model of constant bright-light exposure in female mice to mimic the human situation of artificial light at night, we could not confirm that artificial light is a causal risk factor for atherosclerosis. Further research is necessary to identify whether light at night does in fact contribute to (sex-specific) cardiovascular disease risk in humans.

## Supplemental Material

Supplemental_Material_Journal_of_Biological_Rhythms – Supplemental material for Continuous Light Does Not Affect Atherosclerosis in APOE*3-Leiden.CETP Mice Click here for additional data file. Supplemental material, Supplemental_Material_Journal_of_Biological_Rhythms for Continuous Light Does Not Affect Atherosclerosis in APOE*3-Leiden.CETP Mice by Maaike Schilperoort, Rosa van den Berg, Claudia P. Coomans, Padmini P. S. J. Khedoe, Ashna Ramkisoensing, Sanne Boekestijn, Yanan Wang, Jimmy F. P. Berbée, Johanna H. Meijer, Nienke R. Biermasz, Patrick C. N. Rensen and Sander Kooijman in Journal of Biological Rhythms

## References

[bibr1-0748730420951320] Bedrosian TA Fonken LK Walton JC Nelson RJ (2011) Chronic exposure to dim light at night suppresses immune responses in Siberian hamsters. Biol Lett 7:468-471. 21270021 10.1098/rsbl.2010.1108 PMC3097873

[bibr2-0748730420951320] Bedrosian TA Nelson RJ (2013) Influence of the modern light environment on mood. Mol Psychiatry 18:751-757. 23711982 10.1038/mp.2013.70

[bibr3-0748730420951320] Benjamin EJ Virani SS Callaway CW Chamberlain AM Chang AR Cheng S Chiuve SE Cushman M Delling FN Deo R, et al (2018) Heart disease and stroke statistics—2018 update: a report from the American Heart Association. Circulation 137:e67-e492. 29386200 10.1161/CIR.0000000000000558

[bibr4-0748730420951320] Blume C Garbazza C Spitschan M (2019) Effects of light on human circadian rhythms, sleep and mood. Somnologie (Berl) 23:147-156. 31534436 10.1007/s11818-019-00215-x PMC6751071

[bibr5-0748730420951320] Borbély AA Neuhaus HU (1978) Circadian rhythm of sleep and motor activity in the rat during skeleton photoperiod, continuous darkness and continuous light. J Comp Physiol 128:37–46.

[bibr6-0748730420951320] Brown DL Feskanich D Sanchez BN Rexrode KM Schernhammer ES Lisabeth LD (2009) Rotating night shift work and the risk of ischemic stroke. Am J Epidemiol 169:1370-1377. 19357324 10.1093/aje/kwp056 PMC2727250

[bibr7-0748730420951320] Cappuccio FP Cooper D D’Elia L Strazzullo P Miller MA (2011) Sleep duration predicts cardiovascular outcomes: a systematic review and meta-analysis of prospective studies. Eur Heart J 32:1484-1492. 21300732 10.1093/eurheartj/ehr007

[bibr8-0748730420951320] Chalfant JM Howatt DA Tannock LR Daugherty A Pendergast JS (2020) Circadian disruption with constant light exposure exacerbates atherosclerosis in male ApolipoproteinE-deficient mice. Sci Rep 10:9920. 32555251 10.1038/s41598-020-66834-9 PMC7303111

[bibr9-0748730420951320] Cho CH Lee HJ Yoon HK Kang SG Bok KN Jung KY Kim L Lee EI (2016) Exposure to dim artificial light at night increases REM sleep and awakenings in humans. Chronobiol Int 33:117-123. 26654880 10.3109/07420528.2015.1108980

[bibr10-0748730420951320] Cho CH Yoon HK Kang SG Kim L Lee EI Lee HJ (2018) Impact of exposure to dim light at night on sleep in female and comparison with male subjects. Psychiatry Investig 15:520-530. 10.30773/pi.2018.03.17 PMC5976009 29551048

[bibr11-0748730420951320] Cisse YM Peng J Nelson RJ (2017) Effects of dim light at night on food intake and body mass in developing mice. Front Neurosci 11:294. 28603481 10.3389/fnins.2017.00294 PMC5445163

[bibr12-0748730420951320] Coomans CP van den Berg SA Houben T van Klinken JB van den Berg R Pronk AC Havekes LM Romijn JA van Dijk KW Biermasz NR, et al (2013) Detrimental effects of constant light exposure and high-fat diet on circadian energy metabolism and insulin sensitivity. FASEB J 27:1721-1732. 23303208 10.1096/fj.12-210898

[bibr13-0748730420951320] Druzd D Matveeva O Ince L Harrison U He W Schmal C Herzel H Tsang AH Kawakami N Leliavski A, et al (2017) Lymphocyte circadian clocks control lymph node trafficking and adaptive immune responses. Immunity 46:120-132. 28087238 10.1016/j.immuni.2016.12.011 PMC5263259

[bibr14-0748730420951320] Emmer KM Russart KLG Walker WH Nelson RJ DeVries AC (2018) Effects of light at night on laboratory animals and research outcomes. Behav Neurosci 132:302-314. 29952608 10.1037/bne0000252 PMC6062441

[bibr15-0748730420951320] Fonken LK Lieberman RA Weil ZM Nelson RJ (2013) Dim light at night exaggerates weight gain and inflammation associated with a high-fat diet in male mice. Endocrinology 154:3817-3825. 23861373 10.1210/en.2013-1121

[bibr16-0748730420951320] Fonken LK Nelson RJ (2011) Illuminating the deleterious effects of light at night. F1000 Med Rep 3:18. 21941596 10.3410/M3-18 PMC3169904

[bibr17-0748730420951320] Fonken LK Workman JL Walton JC Weil ZM Morris JS Haim A Nelson RJ (2010) Light at night increases body mass by shifting the time of food intake. Proc Natl Acad Sci USA 107:18664-18669. 20937863 10.1073/pnas.1008734107 PMC2972983

[bibr18-0748730420951320] Garcia-Saenz A Sanchez de Miguel A Espinosa A Valentin A Aragones N Llorca J Amiano P Martin Sanchez V Guevara M Capelo R, et al (2018) Evaluating the association between artificial light-at-night exposure and breast and prostate cancer risk in spain (MCC-Spain Study). Environ Health Perspect 126:047011. 29687979 10.1289/EHP1837 PMC6071739

[bibr19-0748730420951320] Hansson GK Hermansson A (2011) The immune system in atherosclerosis. Nat Immunol 12:204-212. 21321594 10.1038/ni.2001

[bibr20-0748730420951320] Hong J Stubbins RE Smith RR Harvey AE Nunez NP (2009) Differential susceptibility to obesity between male, female and ovariectomized female mice. Nutr J 8:11. 19220919 10.1186/1475-2891-8-11 PMC2650703

[bibr21-0748730420951320] Kloog I Haim A Stevens RG Barchana M Portnov BA (2008) Light at night co-distributes with incident breast but not lung cancer in the female population of Israel. Chronobiol Int 25:65-81. 18293150 10.1080/07420520801921572

[bibr22-0748730420951320] Kloog I Haim A Stevens RG Portnov BA (2009) Global co-distribution of light at night (LAN) and cancers of prostate, colon, and lung in men. Chronobiol Int 26:108-125. 19142761 10.1080/07420520802694020

[bibr23-0748730420951320] Koo YS Song JY Joo EY Lee HJ Lee E Lee SK Jung KY (2016) Outdoor artificial light at night, obesity, and sleep health: cross-sectional analysis in the KoGES study. Chronobiol Int 33:301-314. 26950542 10.3109/07420528.2016.1143480

[bibr24-0748730420951320] Kooijman S van den Berg R Ramkisoensing A Boon MR Kuipers EN Loef M Zonneveld TC Lucassen EA Sips HC Chatzispyrou IA, et al (2015) Prolonged daily light exposure increases body fat mass through attenuation of brown adipose tissue activity. Proc Natl Acad Sci USA 112:6748-6753. 25964318 10.1073/pnas.1504239112 PMC4450411

[bibr25-0748730420951320] Labrecque N Cermakian N (2015) Circadian clocks in the immune system. J Biol Rhythms 30:277-290. 25900041 10.1177/0748730415577723

[bibr26-0748730420951320] Libby P (2002) Inflammation in atherosclerosis. Nature 420:868-874. 12490960 10.1038/nature01323

[bibr27-0748730420951320] Lovejoy JC Sainsbury A (2009) Sex differences in obesity and the regulation of energy homeostasis. Obes Rev 10:154-167. 19021872 10.1111/j.1467-789X.2008.00529.x

[bibr28-0748730420951320] Lucassen EA Coomans CP van Putten M de Kreij SR van Genugten JH Sutorius RP de Rooij KE van der Velde M Verhoeve SL Smit JW, et al (2016) Environmental 24-hr cycles are essential for health. Curr Biol 26:1843-1853. 27426518 10.1016/j.cub.2016.05.038

[bibr29-0748730420951320] McAlpine CS Kiss MG Rattik S He S Vassalli A Valet C Anzai A Chan CT Mindur JE Kahles F, et al (2019) Sleep modulates haematopoiesis and protects against atherosclerosis. Nature 566:383-387. 30760925 10.1038/s41586-019-0948-2 PMC6442744

[bibr30-0748730420951320] McFadden E Jones ME Schoemaker MJ Ashworth A Swerdlow AJ (2014) The relationship between obesity and exposure to light at night: cross-sectional analyses of over 100,000 women in the Breakthrough Generations Study. Am J Epidemiol 180:245-250. 24875371 10.1093/aje/kwu117

[bibr31-0748730420951320] Miller M (2009) Dyslipidemia and cardiovascular risk: the importance of early prevention. QJM 102:657-667. 19498039 10.1093/qjmed/hcp065 PMC2729130

[bibr32-0748730420951320] Obayashi K Saeki K Iwamoto J Ikada Y Kurumatani N (2014) Independent associations of exposure to evening light and nocturnal urinary melatonin excretion with diabetes in the elderly. Chronobiol Int 31:394-400. 24328728 10.3109/07420528.2013.864299

[bibr33-0748730420951320] Obayashi K Saeki K Iwamoto J Okamoto N Tomioka K Nezu S Ikada Y Kurumatani N (2013) Exposure to light at night, nocturnal urinary melatonin excretion, and obesity/dyslipidemia in the elderly: a cross-sectional analysis of the HEIJO-KYO study. J Clin Endocrinol Metab 98:337-344. 23118419 10.1210/jc.2012-2874

[bibr34-0748730420951320] Obayashi K Yamagami Y Tatsumi S Kurumatani N Saeki K (2019) Indoor light pollution and progression of carotid atherosclerosis: a longitudinal study of the HEIJO-KYO cohort. Environ Int 133:105184. 31648154 10.1016/j.envint.2019.105184

[bibr35-0748730420951320] Ohta H Yamazaki S McMahon DG (2005) Constant light desynchronizes mammalian clock neurons. Nat Neurosci 8:267-269. 15746913 10.1038/nn1395

[bibr36-0748730420951320] Orozco-Solis R Aguilar-Arnal L (2020) Circadian regulation of immunity through epigenetic mechanisms. Front Cell Infect Microbiol 10:96. 32232012 10.3389/fcimb.2020.00096 PMC7082642

[bibr37-0748730420951320] Park YM White AJ Jackson CL Weinberg CR Sandler DP (2019) Association of exposure to artificial light at night while sleeping with risk of obesity in women. JAMA Intern Med 179:1061-1071. 10.1001/jamainternmed.2019.0571 PMC6563591 31180469

[bibr38-0748730420951320] Pouwer MG Heinonen SE Behrendt M Andréasson AC van Koppen A Menke AL Pieterman EJ van den Hoek AM Jukema JW Leighton B, et al (2019) The APOE(*)3-Leiden heterozygous glucokinase knockout mouse as novel translational disease model for type 2 diabetes, dyslipidemia, and diabetic atherosclerosis. J Diabetes Res 2019:9727952. 10.1155/2019/9727952 PMC6425338 30949516

[bibr39-0748730420951320] Ramesh V Kaushal N Gozal D (2009) Sleep fragmentation differentially modifies EEG delta power during slow wave sleep in socially isolated and paired mice. Sleep Sci 2:64-75.

[bibr40-0748730420951320] Ramesh V Nair D Zhang SX Hakim F Kaushal N Kayali F Wang Y Li RC Carreras A Gozal D (2012) Disrupted sleep without sleep curtailment induces sleepiness and cognitive dysfunction via the tumor necrosis factor-alpha pathway. J Neuroinflammation 9:91. 22578011 10.1186/1742-2094-9-91 PMC3411474

[bibr41-0748730420951320] Reid KJ Santostasi G Baron KG Wilson J Kang J Zee PC (2014) Timing and intensity of light correlate with body weight in adults. PloS One 9:e92251. 24694994 10.1371/journal.pone.0092251 PMC3973603

[bibr42-0748730420951320] Reutrakul S Knutson KL (2015) Consequences of circadian disruption on cardiometabolic health. Sleep Med Clin 10:455-468. 26568122 10.1016/j.jsmc.2015.07.005 PMC4648711

[bibr43-0748730420951320] Rybnikova NA Haim A Portnov BA (2016) Does artificial light-at-night exposure contribute to the worldwide obesity pandemic? Int J Obes 40:815-823. 10.1038/ijo.2015.255 26795746

[bibr44-0748730420951320] Santhi N Lazar AS McCabe PJ Lo JC Groeger JA Dijk DJ (2016) Sex differences in the circadian regulation of sleep and waking cognition in humans. Proc Natl Acad Sci USA 113:E2730-E2739. 27091961 10.1073/pnas.1521637113 PMC4868418

[bibr45-0748730420951320] Scheiermann C Kunisaki Y Lucas D Chow A Jang JE Zhang D Hashimoto D Merad M Frenette PS (2012) Adrenergic nerves govern circadian leukocyte recruitment to tissues. Immunity 37:290-301. 22863835 10.1016/j.immuni.2012.05.021 PMC3428436

[bibr46-0748730420951320] Schilperoort M van den Berg R Bosmans LA van Os BW Dolle MET Smits NAM Guichelaar T van Baarle D Koemans L Berbee JFP, et al (2020) Disruption of circadian rhythm by alternating light-dark cycles aggravates atherosclerosis development in APOE*3-Leiden.CETP mice. J Pineal Res 68:e12614. 31599473 10.1111/jpi.12614 PMC6916424

[bibr47-0748730420951320] Schipper L Harvey L van der Beek EM van Dijk G (2018) Home alone: a systematic review and meta-analysis on the effects of individual housing on body weight, food intake and visceral fat mass in rodents. Obes Rev 19:614-637. 29334694 10.1111/obr.12663

[bibr48-0748730420951320] Stenzinger M Karpova D Unterrainer C Harenkamp S Wiercinska E Hoerster K Pfeffer M Maronde E Bonig H (2019) Hematopoietic-extrinsic cues dictate circadian redistribution of mature and immature hematopoietic cells in blood and spleen. Cells 8:1033. 10.3390/cells8091033 PMC6769956 31491915

[bibr49-0748730420951320] Stephenson R Lim J Famina S Caron AM Dowse HB (2012) Sleep-wake behavior in the rat: ultradian rhythms in a light-dark cycle and continuous bright light. J Biol Rhythms 27:490-501. 23223374 10.1177/0748730412461247

[bibr50-0748730420951320] van den Maagdenberg AM Hofker MH Krimpenfort PJ de Bruijn I van Vlijmen B van der Boom H Havekes LM Frants RR (1993) Transgenic mice carrying the apolipoprotein E3-Leiden gene exhibit hyperlipoproteinemia. J Biol Chem 268:10540-10545. 7683682

[bibr51-0748730420951320] Vetter C Devore EE Wegrzyn LR Massa J Speizer FE Kawachi I Rosner B Stampfer MJ Schernhammer ES (2016) Association between rotating night shift work and risk of coronary heart disease among women. JAMA 315:1726-1734. 27115377 10.1001/jama.2016.4454 PMC5102147

[bibr52-0748730420951320] Vyas MV Garg AX Iansavichus AV Costella J Donner A Laugsand LE Janszky I Mrkobrada M Parraga G Hackam DG (2012) Shift work and vascular events: systematic review and meta-analysis. BMJ 345:e4800. 22835925 10.1136/bmj.e4800 PMC3406223

[bibr53-0748730420951320] Westerterp M van der Hoogt CC de Haan W Offerman EH Dallinga-Thie GM Jukema JW Havekes LM Rensen PC (2006) Cholesteryl ester transfer protein decreases high-density lipoprotein and severely aggravates atherosclerosis in APOE*3-Leiden mice. Arterioscler Thromb Vasc Biol 26:2552-2559. 16946130 10.1161/01.ATV.0000243925.65265.3c

[bibr54-0748730420951320] Zadelaar AS Boesten LS Jukema JW van Vlijmen BJ Kooistra T Emeis JJ Lundholm E Camejo G Havekes LM (2006) Dual PPARalpha/gamma agonist tesaglitazar reduces atherosclerosis in insulin-resistant and hypercholesterolemic ApoE*3Leiden mice. Arterioscler Thromb Vasc Biol 26:2560-2566. 16931788 10.1161/01.ATV.0000242904.34700.66

