# Supplementary material for: Continuous Light Does Not Affect Atherosclerosis in APOE*3-Leiden.CETP Mice
Source: J Biol Rhythms. 2020 Sep 11;35(6):598–611. doi: 10.1177/0748730420951320 (PMC7683885; doi:10.1177/0748730420951320)
Supplement: Supplemental_Material_Journal_of_Biological_Rhythms – Supplemental material for Continuous Light Does Not Affect Atherosclerosis in APOE*3-Leiden.CETP Mice [file Supplemental_Material_Journal_of_Biological_Rhythms.pdf]

Supplemental Material

Continuous light does not affect atherosclerosis in APOE*3-Leiden.CETP mice

Maaike Schilperoort, Rosa van den Berg, Claudia P. Coomans, Padmini P.S.J. Khedoe, Ashna Ramkisoensing, Sanne Boekestijn, Yanan Wang, Jimmy F.P. Berbée, Johanna H. Meijer, Nienke R. Biermasz, Patrick C.N. Rensen, Sander Kooijman

Supplementary Tables and Figures

Table S1. Primer Sequences for qRT-PCR

Gene	Primer sequence		Product length (bp)
36b4	Forward<br>Reverse	5'– GGACCCGAGAAGACCTCCTT –3'<br>5'– GCACATCACTCAGAATTTCAATGG –3'	85
Bmal1	Forward<br>Reverse	5'– ATGCCAAGACTGGACTTCCG –3'<br>5'– TGCAGAAGCTTTTTCGATCTGC –3'	180
Clock	Forward<br>Reverse	5'– AGTTAGGGCTGAAAGACGGC –3'<br>5'– GGTGTGGAGGAAGGGTCTGA –3'	216
Cry1	Forward<br>Reverse	5'– AGAGGGCTAGGTCTTCTCGC –3'<br>5'– GTGAGTCTGCTGACTGTCCC –3'	222
Cry2	Forward<br>Reverse	5'– CCAGAGCACTATCCAGTGGC –3'<br>5'– GCGATGGCTCTCCAGTCTC –3'	154
Per1	Forward<br>Reverse	5'– ACGGCCAGGTGTCGTGATTA –3'<br>5'– CCCTTCTAGGGGACCACTCA –3'	162
Per2	Forward<br>Reverse	5'– TGTGTGCTTACACGGGTGTCCTA –3'<br>5'– ACGTTTGGTTTGCGCATGAA –3'	142
Reverba	Forward<br>Reverse	5'– GTGCTTGTCTCTGCAGACCG –3'<br>5'– TTGGTGAAGCGGGAAGTCTC –3'	131

Table S2. Values of statistical testing by two-tailed unpaired T test

**Parameter**	**T-value**	**Degrees of freedom (df)**	**P-value**
Food intake	2.442	34	**0.0200**
Fat mass	**1.052**	33	0.3006
Lean mass	1.342	33	0.1887
Organ weight - Liver	0.662	34	0.5126
Organ weight – Spleen	1.718	34	0.0952
Organ weight – iBAT	1.649	34	0.1083
Organ weight – gWAT	1.190	34	0.2422
Organ weight – sWAT	0.747	34	0.4600
Cholesterol exposure	2.013	34	0.0521
Bone marrow WBCs	2.765	33	**0.0092**
Bone marrow RBCs	2.821	34	**0.0079**
Bone marrow PLTs	1.742	34	0.0905
Blood WBCs	4.007	34	**0.0003**
Blood RBCs	0.659	34	0.5142
Blood PLTs	2.610	34	**0.0134**
Blood neutrophils	1.507	28	0.1430
Blood lymphocytes	0.894	29	0.3787
Blood monocytes	0.456	29	0.6518
Blood eosinophils	0.108	29	0.9145
Blood basophils	2.204	34	**0.0344**
Atherosclerotic lesion area	0.620	33	0.5396
Lesion severity - mild	1.552	32	0.1302
Lesion severity - severe	1.550	32	0.1309
Rhythm strength	2.455	24	**0.0217**
Rhythm period	10.26	24	**<0.0001**
Clock expression in iBAT	0.381	16	0.7082
Bmal1 expression in iBAT	2.133	16	**0.0487**
Reverba expression in iBAT	4.514	16	**0.0004**
Cry1 expression in iBAT	3.541	16	**0.0027**
Cry2 expression in iBAT	2.330	16	**0.0332**
Per1 expression in iBAT	0.709	16	0.4883
Per2 expression in iBAT	3.005	16	**0.0084**
Clock expression in gWAT	2.241	16	**0.0396**
Bmal1 expression in gWAT	0.017	16	0.9870
Reverba expression in gWAT	3.606	16	**0.0024**

Cry1 expression in gWAT	1.080	16	0.2961
Cry2 expression in gWAT	1.140	16	0.2711
Per1 expression in gWAT	0.645	16	0.5278
Per2 expression in gWAT	1.921	16	0.0728
Clock expression in liver	0.856	16	0.4044
Bmal1 expression in liver	0.721	16	0.4815
Reverba expression in liver	2.825	16	**0.0122**
Cry1 expression in liver	1.532	16	0.1452
Cry2 expression in liver	1.391	16	0.1831
Per1 expression in liver	1.087	16	0.2932
Per2 expression in liver	0.514	16	0.6146
Clock expression in aorta	0.846	15	0.4109
Bmal1 expression in aorta	1.021	15	0.3236
Reverba expression in aorta	3.198	15	**0.0060**
Cry1 expression in aorta	1.821	15	0.0886
Cry2 expression in aorta	0.248	15	0.8076
Per1 expression in aorta	0.983	15	0.3410
Per2 expression in aorta	2.364	15	**0.0320**

P-values are marked bold when statistically significant (P<0.05).

Table S3. Values of statistical testing by ANOVA or mixed-effects models

**Parameter**	**Statistical test including group, time and group*time interaction effects**	**Adjusted P-values of Sidak's post hoc test**
Cumulative food intake	Two-way ANOVA<br>Group F (1, 6) = 1.563; P=0.258<br>Time F (1.260, 7.559) = 1462; **P<0.0001**<br>Interaction F (10, 60) = 2.253; **P=0.026**	T≈1 week >0.999<br>T≈3 weeks >0.999<br>T≈4 weeks 0.996<br>T≈5 weeks 0.979<br>T≈6 weeks 0.962<br>T≈7 weeks 0.901<br>T≈8 weeks 0.887<br>T≈9 weeks 0.924<br>T≈10 weeks 0.931<br>T≈11 weeks 0.975<br>T≈12 weeks 0.984

Body weight	<u>Two-way ANOVA</u><br>Group F (1, 34) = 0.007; P=0.934<br>Time F (2.752, 93.55) = 137.2; **P<0.0001**<br>Interaction F (5, 170) = 3.502; **P=0.005**	T=0 weeks<br>T=2 weeks<br>T=4 weeks<br>T=8 weeks<br>T=12 weeks<br>T=14 weeks	0.782<br>>0.999<br>>0.999<br>0.989<br>0.865<br>0.996
Triglycerides	<u>Mixed-effects models</u><br>Group F (1, 34) = 2.028; P=0.164<br>Time F (5, 162) = 16.49; **P<0.0001**<br>Interaction F (5, 162) = 2.101; P=0.068	T=0 weeks<br>T=2 weeks<br>T=4 weeks<br>T=8 weeks<br>T=12 weeks<br>T=14 weeks	0.999<br>0.848<br>0.057<br>0.909<br>0.985<br>0.501
Total cholesterol	<u>Mixed-effects models</u><br>Group F (1, 34) = 4.042; P=0.052<br>Time F (3.160, 99.84) = 44.11; **P<0.0001**<br>Interaction F (5, 158) = 2.801; **P=0.019**	T=0 weeks<br>T=2 weeks<br>T=4 weeks<br>T=8 weeks<br>T=12 weeks<br>T=14 weeks	0.999<br>0.583<br>0.234<br>0.379<br>0.205<br>0.954
HDL cholesterol	<u>Mixed-effects models</u><br>Group F (1,33) = 0.1119; P=0.740<br>Time F (2, 58) = 8.369; **P=0.0006**<br>Interaction F (2, 58) = 0.967; P=0.386	T=8 weeks<br>T=12 weeks<br>T=14 weeks	0.930<br>0.807<br>0.765
Non-HDL cholesterol	<u>Mixed-effects models</u><br>Group F (1,33) = 5.771; **P=0.022**<br>Time F (1.936, 49.36) = 20.52; **P<0.0001**<br>Interaction F (2, 51) = 1.506; P=0.2315	T=8 weeks<br>T=12 weeks<br>T=14 weeks	0.219<br>**0.038**<br>0.542
Rhythm strength per day (LD vs. LD-DL)	<u>Two-way ANOVA</u><br>Group F (1, 11) = 1.238; P=0.290<br>Time F (1.409, 15.50) = 14.17; **P=0.0008**<br>Interaction F (9, 99) = 11.89; **P<0.0001**	T=95 days<br>T=96 days<br>T=97 days<br>T=98 days<br>T=99 days<br>T=100 days<br>T=101 days<br>T=102 days<br>T=103 days<br>T=104 days	>0.999<br>>0.999<br>>0.999<br>0.172<br>**0.003**<br>0.179<br>0.938<br>0.974<br>>0.999<br>>0.999

P-values are marked bold when statistically significant (P<0.05).

Table S4. Values of Pearson correlation analyses

Correlation	Group	R-squared	95% Confidence interval	P-value
Atherosclerotic lesion area with rhythm strength	LD&LL<br>LD<br>LL	0.0372<br>0.3956<br>0.3565	-0.2189 to 0.5464<br>-0.9241 to 0.1360<br>-0.1499 to 0.7147	0.3555<br>0.0948<br>0.1602
Severe atherosclerotic lesions with rhythm strength	LD&LL<br>LD<br>LL	0.00044<br>0.02863<br>0.00012	-0.3856 to 0.4209<br>-0.6079 to 0.7808<br>-0.5039 to 0.4874	0.9221<br>0.6887<br>0.9679

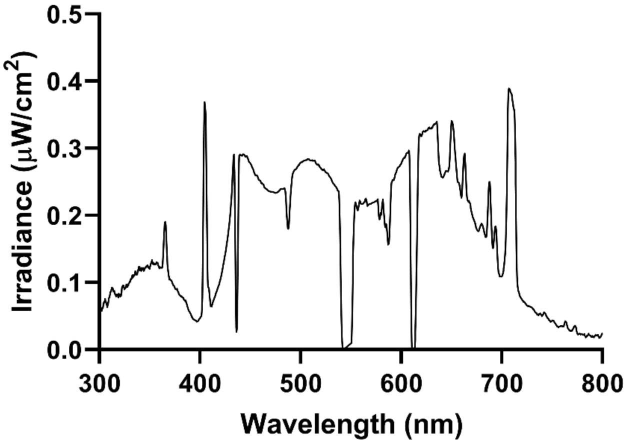

Figure S1. Spectral power distribution of the light source. Mice were housed in light-tight cabinets fitted with diffuse white fluorescent light of ~100 lux. The spectral power distribution of the light source was measured with an AvaSpec 2048-SPU (Avantes BV, Apeldoorn, The Netherlands) light meter. Adapted from Schilperoort *et al*, J Pineal Res, 2020 (DOI:10.1111/jpi.12614).

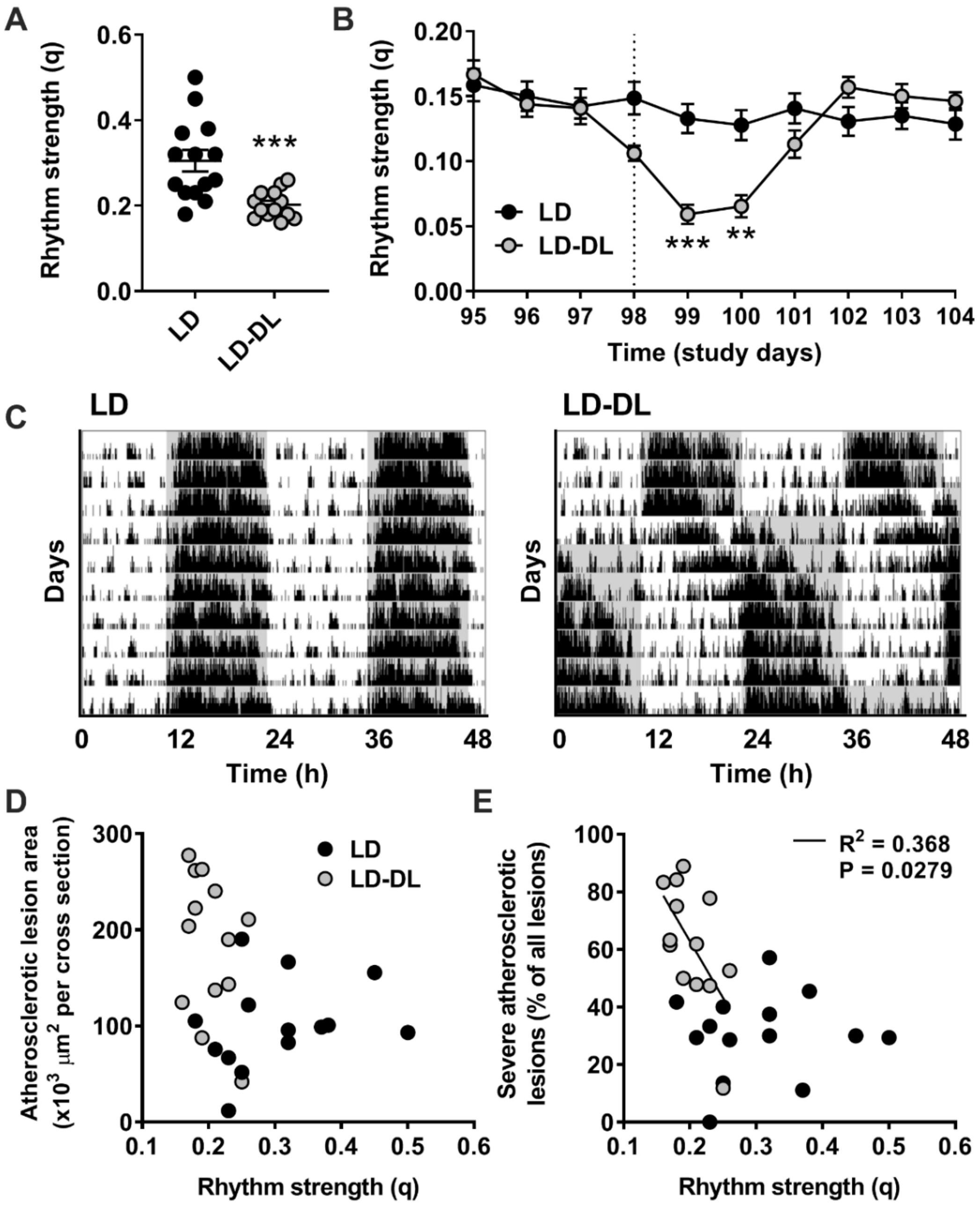

Figure S2. Shifts in light-dark cycle promote a strong acute reduction in rhythm strength, which correlates to atherosclerotic lesion severity. APOE*3-Leiden.CETP mice fed a Western-type diet were exposed to either regular light-dark cycles (LD) or weekly alternating light-dark cycles (12 h shifts; LD-DL) (*n* = 15/group) for 15 weeks. During week 14 and 15 of the light intervention, mice were housed individually in cages fitted with passive infrared detectors to assess behavioral activity patterns. F-periodogram analysis was performed to calculate the rhythm strength per week (A) and per day (B), and the dotted line in figure panel B indicates the day on which the LD cycle is shifted for the LD-DL group. Representative double-plotted actograms of LD and LL mice are shown (C). Rhythm strength was correlated to the atherosclerotic lesion area (D) and the relative amount of severe atherosclerotic lesions (E), and the R-squared and P-values are shown for the significant correlation between rhythm strength and severe atherosclerotic lesions within the LD-DL group, as evaluated by Pearson correlation analysis. Data are expressed as individual values or as means ± SEM. Significance was tested by the two-tailed unpaired Student T test (A) or mixed models (B). **P<0.01, ***P<0.001 compared to the LD control group.

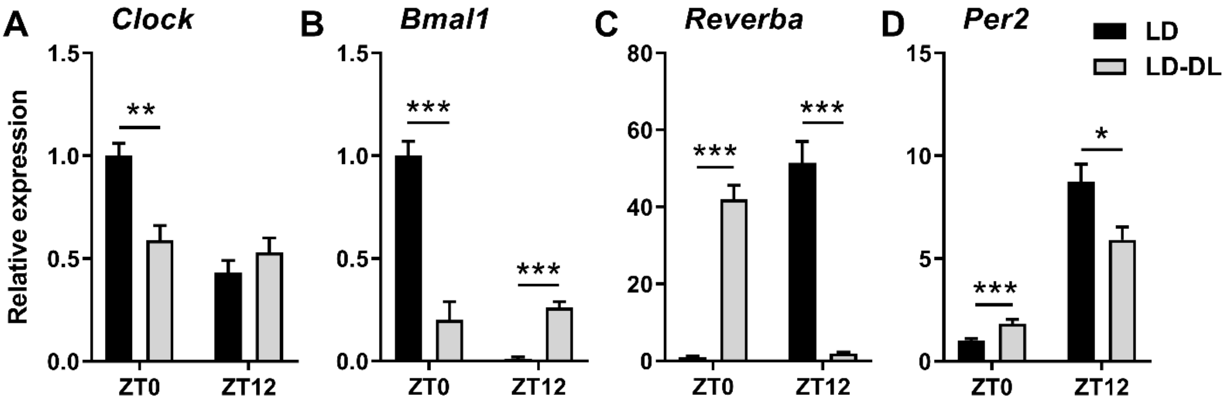

Figure S3. Expression of clock genes in the aorta is disrupted in mice exposed to shifts in light-dark cycle. APOE*3-Leiden.CETP mice fed a Western-type diet were exposed to regular light-dark cycles (LD) or weekly alternating light-dark cycles (LD-DL) (*n* = 18/group) for 10 weeks, after which aortas were isolated at either ZT0 or ZT12 (*n* = 7-8 per timepoint/group) for gene expression analysis of *Clock* (A), *Bmal1* (B), *Reverba* (C), and *Per2* (D). Data are expressed as means ± SEM. Significance was tested by two-way ANOVA. *P<0.05, **P<0.01, ***P<0.001 compared to the LD control group.

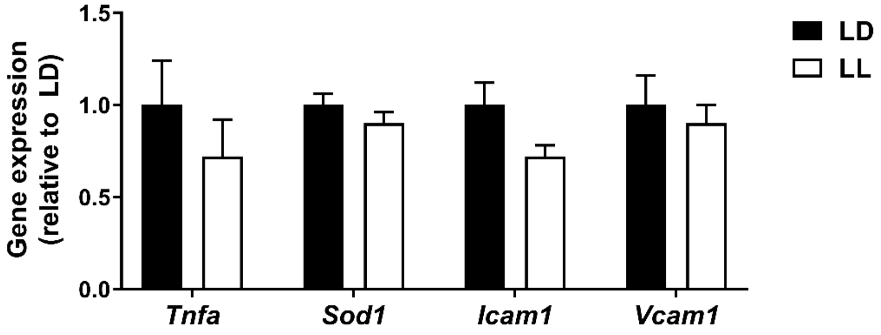

Figure S4. Expression of markers of inflammation, oxidative stress and leukocyte recruitment in the aorta is unaffected by exposure to constant light. APOE*3-Leiden.CETP mice fed a Western-type diet were exposed to LD or LL (*n* = 18/group) for 14 weeks, after which aortas were isolated (*n* = 9/group) at ZT2 for gene expression analysis of markers of inflammation (*Tnfa*), oxidative stress (*Sod1*), and leukocyte recruitment (*Icam1* and *Vcam1*). Data are expressed as means ± SEM. Significance was tested by the two-tailed unpaired Student T test.
